# Larval morphology of *Phratora koreana* Takizawa, 1985 with a key to the larvae of the Palaearctic *Phratora* species (Coleoptera, Chrysomelidae, Chrysomelinae)

**DOI:** 10.3897/zookeys.658.11068

**Published:** 2017-02-23

**Authors:** Hee-Wook Cho, Jolanta Świętojańska

**Affiliations:** 1 Department of Biodiversity and Evolutionary Taxonomy, University of Wrocław, Przybyszewskiego 65, 51-148 Wrocław, Poland

**Keywords:** First and third instar larvae, leaf beetles, life cycle, morphology, South Korea

## Abstract

The first and third instar larvae of *Phratora koreana* Takizawa, 1985 are described and illustrated in detail for the first time. Morphological changes in the pigmentation, tubercular pattern and defensive glands during the larval development are discussed. The life cycle and host-plant of *Phratora koreana* and a key to the larvae of the Palaearctic *Phratora* species are also provided.

## Introduction

The genus *Phratora* Chevrolat, 1836 is widely distributed in the Holarctic region, and also in the Oriental region restricted to montane areas (Ge et al. 2004). In the Palaearctic region, the genus is represented by 32 species (Kippenberg 2010), and larval morphology has been known for nine species (Kimoto and Takizawa 1994, Steinhausen 1994, Zaitzev and Medvedev 2009, etc.). Both adult and larva feed on the leaves of *Salix*, *Populus* and *Betula* (Jolivet and Hawkeswood 1995) and include several important pests, such as *Phyllodecta vitellinae* (Linnaeus, 1758) and *Phyllodecta vulgatissima* (Linnaeus, 1758) (Batley et al. 2004). Based on the larval and pupal morphology and biology, the genus *Phratora* belongs to the generic group *Phaedon* proposed by Kimoto (1962) with the genera *Phaedon* Latreille, *Gastrophysa* Chevrolat and *Mesoplatys* Baly and is easily distinguished by the presence of tubercles Dai and Dp on abdominal segment I in the third instar larva (Takizawa 1976).

*Phratora koreana* was described from South Korea by Takizawa (1985). Later (1990) he reported it from Japan, but nothing is so far known about its immature stages, life cycle and host-plants. The first author collected the adult and egg of *Phratora koreana* from Mt. Hambaeksan in South Korea, and larvae were obtained from eggs. Here the first and third instar larvae of *Phratora koreana* are described in detail for the first time. Morphological changes during the larval development are discussed, with a key to the known mature larvae of the Palaearctic *Phratora*.

## Materials and methods

Eggs were collected along with adults on the host-plant *Salix caprea* on 6 June 2006 in South Korea, Gangwon Province, Taebaek-si, Mt. Hambaeksan, 37°16.30'N; 128°91.75'E, ca 1500 m. Larvae were reared from eggs in plastic containers (10 cm diameter, 12 cm deep), and then preserved in 70% ethanol. For examination of morphological characters, some larvae were dissected, cleared in 10% sodium hydroxide solution, rinsed in distilled water, and then mounted on slides with glycerine and Swan’s liquid (20 g distilled water, 15 g gum arabic, 60 g chlorhydrate, 3 g glucose and 2 g glacial acetic acid). Descriptions and illustrations were prepared using a Nikon SMZ800 stereomicroscope and a Nikon ECLIPSE 80i light microscope with phase contrast, each microscope equipped with a camera lucida. Photographs were taken by a Nikon D5200 digital camera attached to a Nikon SMZ18 microscope, and were edited in Helicon Focus 5.3.12 and Adobe Photoshop CS5. The specimens were deposited in the Department of Biodiversity and Evolutionary Taxonomy, University of Wrocław, Poland and H.-W. Cho’s private collection, South Korea. The terminology of the larval tubercles follows Kimoto (1962). The letters L, S and M in parentheses signify long, short, and minute setae, respectively.

## Systematics

### Phratora koreana

Taxon classificationAnimaliaColeopteraChrysomelidae

Takizawa, 1985

Figs 1–3 , 4–5 , 6–14 , 15–16

#### Description of the larva.

**First instar larva.** Body length 2.12–2.38 mm, width 0.56–0.62 mm, head width 0.42–0.44 mm (n = 3). Body yellowish-white with head brown, tubercles and legs light brown in alcohol; integument moderately covered with sclerotized platelets. Defensive glands very large on meso- and metathorax, but almost invisible on abdominal segments I–VII. Egg bursters present on meso- and metathorax. Head and mouthparts similar in the shape and chaetotaxy to those of the third instar larva.

*Thorax*. Prothorax with D-DL-EPa (2–3L 5–6S 2–3M) entirely pigmented; EPp (1S); P (1M); ES-SS (2M) weakly sclerotized (Fig. 15). Meso- and metathorax with Da (2S); Dp (1S); DLi (1S 2M) with a small egg burster situated anterior to a short seta; DLe (2L 2M) conical with a large defensive gland; EPa (1S); EPp (1S); P (1M); SS (1M) and ES (1M) weakly sclerotized.

*Abdomen*. Segments I–VI with Dai (1S); Dp (2S 1M); DL (1L 2S); EP (1L 1S 0–1M); P (1S 1M); PS-SS (3M) divided into two tubercles; ES (1M) on both sides generally fused. Segment VII with dorsal tubercles enlarged and fused. Segments VIII–IX each with dorsal and dorso-lateral tubercles enlarged and fused. Segment X with pygopod well developed.

**Figures 1–3.**
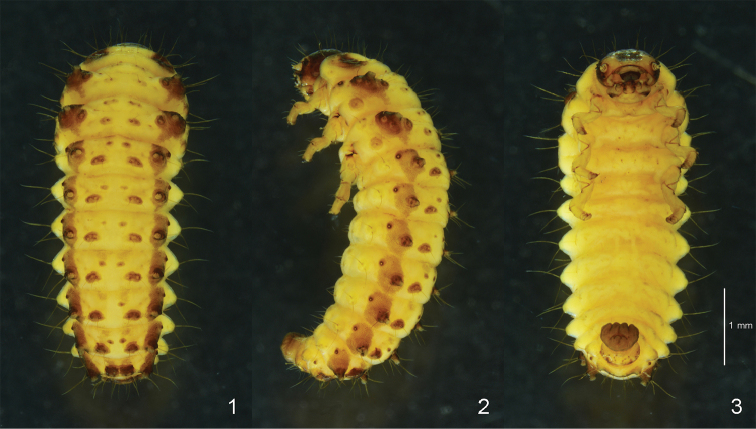
Habitus of *Phratora koreana*, third instar larva. **1** dorsal **2** lateral **3** ventral.

**Third (last) instar larva.** Body length 4.60–5.80 mm, width 1.50–1.90 mm, head width 0.85–0.90 mm (n = 7). Body elongate, rather broad, widest at meso- and metathorax, thence moderately narrowed posteriorly (Fig. 1) and moderately convex dorsally (Fig. 2). General coloration of integument yellowish-white in alcohol; dorso-lateral region covered with dense platelets, forming a pair of longitudinal bands; head dark brown with anterior region and mouthparts largely yellowish-white; dorsal tubercles small, pale brown to dark brown, whereas ventral ones reduced (Fig. 3); legs pale yellow with apex of each segment brown. Defensive glands present on meso- and metathorax and abdominal segments I–VII.

*Head*. Hypognathous, rounded, strongly sclerotized (Fig. 6). Vertex with four pairs of minute setae; epicranium with six pairs of long setae; temporal side of head with three pairs of long setae. Epicranial suture distinct; frontal suture short, not reaching antennal socket; endocarina well developed. Frons with four pairs of long setae. Clypeus trapezoidal with two pairs of setae. Labrum slightly emarginate with two pairs of setae and two pairs of campaniform sensilla placed medially and one pair of setae placed at anterior border (Fig. 11); epipharynx with four pairs of stout and four pairs of small setae at anterior margin and one pair of small setae placed medially (Fig. 12). Mandible palmate, 4-toothed, with two setae and two campaniform sensilla (Figs 7–8). Maxillary palp 3-segmented; palpomere I transverse with one seta and one campaniform sensillum; II rectangular with two setae and one campaniform sensillum; III subconical with one seta, one digitiform sensillum and one campaniform sensillum on sides and a group of peg-like sensilla at the apex; palpifer distinct with two setae (Fig. 13). Mala rounded with eight pointed setae, two blunt setae and one campaniform sensillum; stipes longer than wide with three setae; cardo without setae. Labial palp 2-segmented; palpomere I rectangular with singe campaniform sensillum; II subconical with one campaniform sensillum below the apex and a group of peg-like sensilla at the apex. Hypopharyngeal area with three pairs of short setae and two pairs of campaniform sensilla. Prementum with two pairs of short setae and one pair of small setae – each of them placed at base of labial palp; postmentum with three pairs of setae. Stemmata six on each side, four of them located above antenna and two behind antenna. Antenna short, 3-segmented; antenomere I transverse with four campaniform sensilla; II stout, more or less as wide as long, with a conical sensorium and five small setae apically; III subconical with six small setae apically (Figs 9–10).

*Thorax*. Prothorax with D-DL-EPa (8–9L 2–3S) largest and pigmented only on dorso-lateral region; EPp (1S); P (1S) not sclerotized; ES-SS represented by a short seta (Fig. 16). Meso- and metathorax with Da (2S) small and transverse; Dp (1L) subequal to Da in size; DLi (1L 2S) larger than Da and Dp; DLe (2L 2–3S 1M) large and conical with a defensive gland; EPa (1L); EPp (1L); P (1S) not sclerotized; SS (1S) and ES (1S) represented by setae. Mesothoracic spiracles annuliform; peritreme fused with EPa. Legs rather stout; tibia with nine setae; tarsungulus large, strongly curved, basal tooth not developed, with 1 short seta (Fig. 14).

*Abdomen*. Segments I–VI with Dai (1S) very small; Dp (2L 1S) small, but larger than Dai; DL (2L 1M) conical with a defensive gland; EP (2L) and P (2S) not sclerotized; PS-SS (3S) and ES (1S) represented by setae. Segment VII with dorsal tubercles enlarged and fused; DL with a defensive gland. Segments VIII–IX each with dorsal and dorso-lateral tubercles fused. Segment X with pygopod well developed. Spiracles present on segments I–VIII.

**Figures 4–5.**
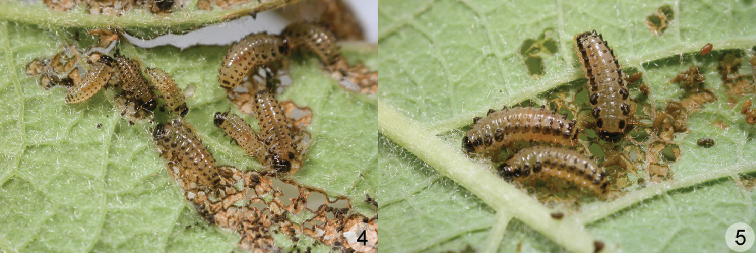
*Phratora koreana*, live larva. **4** first and second instar larvae **5** third instar larva.

**Figures 6–14.**
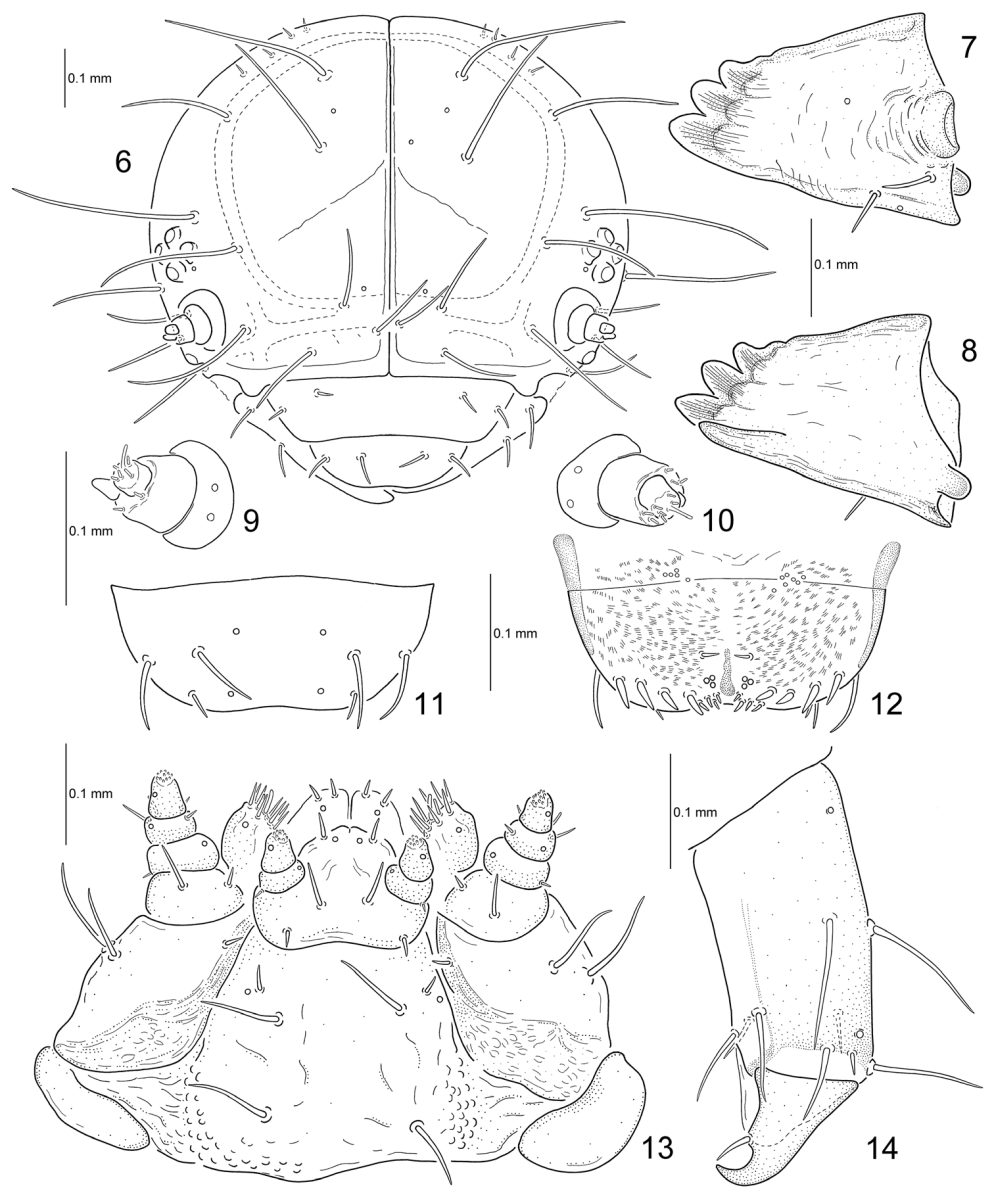
*Phratora koreana*, third instar larva. **6** head **7–8** mandibles **9–10** antennae **11** labrum **12** epipharynx **13** maxillae and labium **14** tibia and tarsungulus.

**Figures 15–16.**
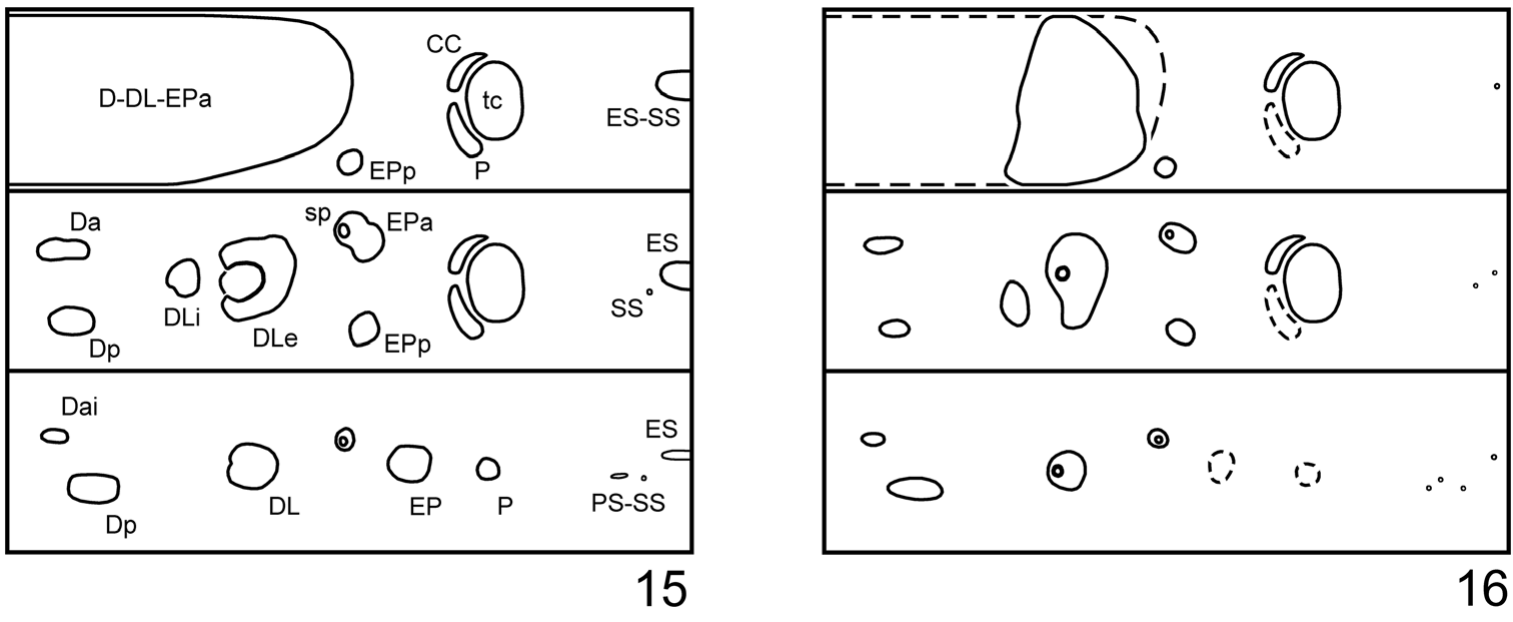
Schematic presentation of tubercular patterns (top: prothorax, middle: mesothorax, bottom: abdominal segment II), *Phratora koreana*. **15** first instar larva **16** third instar larva.

#### Diagnosis.

The larva of *Phratora koreana* is easily distinguished from all other known species of *Phratora* by the presence of small tubercles Dai and Dp on abdominal segments I–VI. In other species of *Phratora*, Dai and Dp are present only on abdominal segment I and a large tubercle D is present on II–VI. The larva of *Prasocuris glabra* (Herbst) is also similar to that of *Phratora koreana* in the presence of tubercles Dai and Dp on abdominal segments I–VI, but tubercles of *Prasocuris glabra* are much larger (Hennig 1938).

#### Distribution.

South Korea: Gangwon, Gyeongnam, Jeju; Japan: Honshu (Takizawa 1985, 1990).

#### Notes on biology and larval morphology.

Overwintered adults appear in late May, mate and lay 8–15 yellowish eggs per cluster on leaves of *Salix caprea* in early June. The larvae gregariously feed on leaves until the final instar. There are three larval instars and pupation takes place in the soil. Newly emerged adults appear in early July.

Morphological changes in the pigmentation, tubercular pattern and defensive glands occur during the larval development. The first instar larva has well developed and pigmented tubercles, but after molting to the second instar larva, ventral tubercles are reduced and median region of D-DL-EPa is unpigmented (Fig. 4). The defensive glands on abdominal segments I–VII are well developed in the second and third instar larvae, whereas they are almost invisible in the first instar larva. A pair of longitudinal bands on account of very dense and strongly sclerotized platelets appear only in the third instar larva (Fig. 5).

##### Key to the known third instar larvae of the Palaearctic species of the genus *Phratora*

(modified from Steinhausen 1994, Zaitzev and Medvedev 2009)

**Table:** 

1	Claws with basal tooth (subgenus Phratora s.str.)	**2**
–	Claws without basal tooth (subgenus Phyllodecta Kirby)	**3**
2	Claws with large and quadrangular basal tooth; dorsal coloration mostly “pale” or “dark”, rarely “striped”. Forest belt of Palaearctic	***vulgatissima* (Linnaeus)**
–	Claws with long, narrow and sharp basal tooth; dorsal coloration mostly “striped”. East Siberia, Far East	***obtusicollis* Motschulsky**
3	The underside of the body with unpigmented and hardly visible tubercles	**4**
–	The underside of the body with pigmented and distinct tubercles	**6**
4	Tubercles Dai and Dp present on abdominal segments II–VI. Korea and Japan	***koreana* Takizawa**
–	Tubercle D present on abdominal segments II–VI	**5**
5	Dorsal coloration mostly “dark”, rarely “pale” or “striped”; tubercle EP unpigmented. Forest belt of Palaearctic	***laticollis* (Suffrian)**
–	Dorsal coloration “dark”; tubercle EP pigmented. Taiwan	***similis* (Chûjô)**
6	Body covered with black setae, which are usually darker than the rest of surface; dorsal side of the body covered unevenly with microsculpture, forming separate dark spots between tubercles; pronotum dark with 2 yellow spots near mid-line; body wide, narrowed posteriorly, depressed dorsally, with head distinctly narrower than thorax. Forest belt of Palaearctic	***vitellinae* (Linnaeus)**
–	Body covered with pale setae, which are usually not darker than the rest of surface, or setae paler than the rest of surface; dorsal side of the body covered with dense and even microsculpture, not forming separate spots; dorsal side giving dark impression; body elongate, cylindrical, with head indistinctly narrower than thorax	**7**
7	Pronotum black with narrow pale medial stripe. Central Europe, European Russia, North Caucasus	***tibialis* (Suffrian)**
–	Pronotum pale medially	**8**
8	Pronotum black with wide pale stripe occupying medial 1/3. Central Europe, Northern Europe, North of European Russia, Siberia, Far East, Arctic	***polaris* (Schneider)**
–	Pronotum pale with dark brown lateral sides. Forest belt of Palaearctic	***atrovirens* (Cornelius)**

**Notes**. *Phratora grandis* (Chûjô, 1956) occurring in Japan is not included in the key due to insufficient description. This species differs from other Japanese species in having black head and legs with all tubercles dark brown (Kimoto and Takizawa 1994).

## Supplementary Material

XML Treatment for Phratora koreana
